# Nicotine chewing gum for the prevention of postoperative ileus after colorectal surgery: a multicenter, double-blind, randomised, controlled pilot study

**DOI:** 10.1007/s00384-017-2839-z

**Published:** 2017-06-28

**Authors:** Daniël P. V. Lambrichts, Geesien S. A. Boersema, Buket Tas, Zhouqiao Wu, Wietske W. Vrijland, Gert-Jan Kleinrensink, Johannes Jeekel, Johan F. Lange, Anand G. Menon

**Affiliations:** 1 Department of Surgery, Erasmus University Medical Center, Room H822k, PO BOX 2040, 3000 CA Rotterdam, The Netherlands; 2 Key Laboratory of Carcinogenesis and Translational Research (Ministry of Education, Beijing), Ward I of Gastrointestinal Cancer Center, Peking University Cancer Hospital & Institute, Beijing, China; 3 Department of Surgery, Franciscus Gasthuis & Vlietland, Rotterdam, The Netherlands; 4 Department of Neuroscience, Erasmus University Medical Center, Rotterdam, The Netherlands; 5 Department of Surgery, Havenziekenhuis, Rotterdam, The Netherlands

**Keywords:** Postoperative ileus, Prevention, Nicotine chewing gum, Colorectal surgery

## Abstract

**Purpose:**

When postoperative ileus is not resolved after 5 days or recurs after resolution, prolonged POI (PPOI) is diagnosed. PPOI increases discomfort, morbidity and hospitalisation length, and is mainly caused by an inflammatory response following intestinal manipulation. This response can be weakened by targeting the cholinergic anti-inflammatory pathway, with nicotine as essential regulator. Chewing gum, already known to stimulate gastrointestinal motility itself, combined with nicotine is hypothesised to improve gastrointestinal recovery and prevent PPOI. This pilot study is the first to assess efficacy and safety of nicotine gum in colorectal surgery.

**Methods:**

Patients undergoing elective oncological colorectal surgery were enrolled in this double-blind, parallel-group, controlled trial and randomly assigned to a treatment protocol with normal or nicotine gum (2 mg). Patient reported outcomes (PROMS), clinical characteristics and blood samples were collected. Primary endpoint was defined as time to first passage of faeces and toleration of solid food for at least 24 h.

**Results:**

In total, 40 patients were enrolled (20 vs. 20). In both groups, six patients developed PPOI. Time to primary endpoint (4.50 [3.00–7.25] vs. 3.50 days [3.00–4.25], *p* = 0.398) and length of stay (5.50 [4.00–8.50] vs. 4.50 days [4.00–6.00], *p* = 0.738) did not differ significantly between normal and nicotine gum. There were no differences in PROMS, inflammatory parameters and postoperative complications.

**Conclusions:**

We proved nicotine gum to be safe but ineffective in improving gastrointestinal recovery and prevention of PPOI after colorectal surgery. Other dosages and administration routes of nicotine should be tested in future research.

**Electronic supplementary material:**

The online version of this article (doi:10.1007/s00384-017-2839-z) contains supplementary material, which is available to authorized users.

## Introduction

Postoperative ileus (POI) is a temporary inhibition of gastrointestinal motility after abdominal surgery and is usually associated with nausea, vomiting, abdominal distension and lack of flatus and defaecation [1, 2]. In more than 50% of cases, POI is not fully resolved in 4 days after the operation and when it does not resolve after 5 days or recurs after an apparent resolution, prolonged POI (PPOI) is diagnosed [3, 4]. PPOI causes an increase in patient discomfort, morbidity, hospital-acquired infections, hospitalisation days and healthcare costs [5].

The aetiology of POI is complex, with multiple factors contributing to its pathogenesis [6]. Opioid use for postoperative analgesia is known to inhibit gastrointestinal transit and prolong POI [7, 8]. However, the development of POI after abdominal surgery is mainly caused by intestinal manipulation during the surgical procedure, thereby triggering an inflammatory response and causing a sustained and generalised gastrointestinal hypomotility [7, 9, 10]. Targeting this inflammatory response is of clinical relevance, but effective strategies are not yet available in clinical practice [8].

The cholinergic anti-inflammatory pathway (CAIP) is one of the mechanisms that can be targeted for the prevention of POI. Experimental studies have shown that mediation of CAIP by vagus nerve stimulation can increase bowel motility and control inflammatory cell recruitment, by that preventing pathological changes important in the development of POI [1, 8, 11]. Moreover, nicotinic acetylcholine receptors (nAChR) play an important role in mediation of CAIP, making nicotine an essential regulator of the pathway [11–13]. Additionally, the α7-nAChR also plays a role in nicotine-induced analgesia [14] and clinical evidence shows that preoperative transdermal and intranasal administration of nicotine significantly reduced postoperative opioid use [15, 16], while reducing opioids is an important strategy of shortening POI [1, 8, 17].

**Fig. 1:**
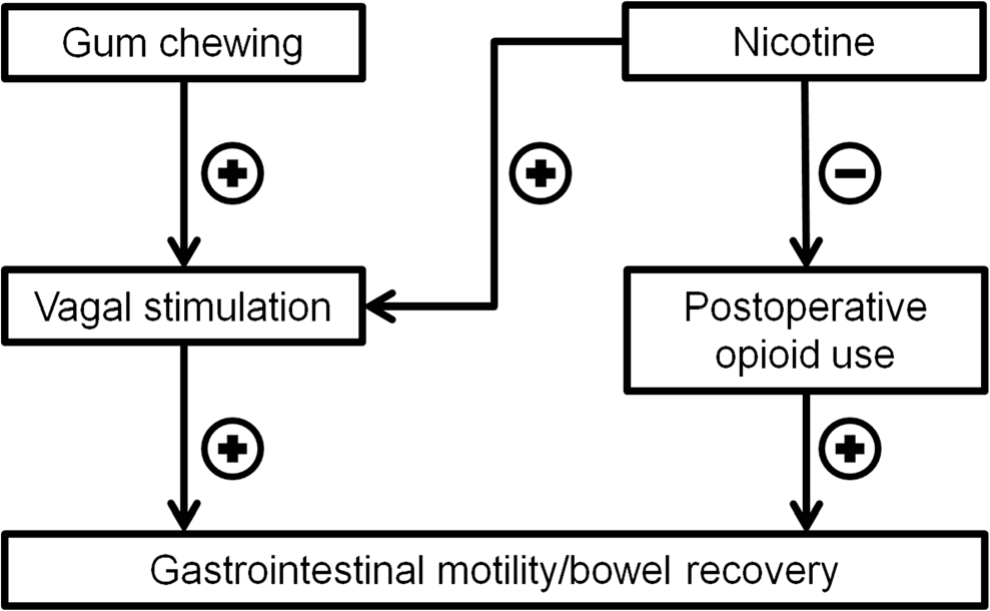
Simplified scheme of hypothesised effect mechanism of nicotine chewing gum

Gum chewing is another important strategy, which has already been proven to be beneficial for gastrointestinal recovery after surgery. Several systematic reviews and meta-analyses have been published, supporting postoperative gum chewing in abdominal surgery [18–23]. As a form of sham feeding, it mimics the cephalic phase of digestion and stimulates the gastrointestinal motility via neurohormonal and vagal pathways [18, 24]. Combining perioperative gum chewing with the potential beneficial effects of nicotine leads to the hypothesis that nicotine gum chewing can reduce POI and improve postoperative outcomes (e.g. less morbidity and shorter length of stay) as well as reduce medical costs [25]. The commercially available and inexpensive nicotine chewing gum may have a wide clinical application in POI prevention, by both stimulating the cephalic-vagal reflex and activating CAIP (Fig. 1). Therefore, we performed a multicenter, randomised, double-blind, controlled pilot study, comparing perioperative use of nicotine chewing gum with normal chewing gum, to assess the clinical efficacy and safety in patients undergoing colorectal surgery.

## Methods

### Study design and participants

This is a prospective, parallel-group, double-blind, randomised, controlled pilot study, conducted in the Havenziekenhuis, Rotterdam and the Sint Franciscus Gasthuis, Rotterdam. Adult patients who underwent an elective oncologic colorectal resection and gave written consent were included. Exclusion criteria were severe chronic cardiovascular disease or acute cardiovascular disease, severe liver- or kidney disease, oral or pharyngeal infection, esophagitis, hypersensitivity to any component of the nicotine gum, previous colorectal surgery, pregnancy, breast feeding, having an elevated risk of choking or being unable to chew gum for any reason.

### Study procedures

Patients received either normal chewing gum or Nicorette® 2-mg chewing gum (2 mg/gum). This nicotine chewing gum is normally used as nicotine replacement therapy to help control craving for cigarettes and contains a low dose of nicotine. Patients had to chew the allocated chewing gum 2 h preoperatively and three times a day postoperatively, for half an hour at a time, until the first passage of faeces and tolerance of solid food for more than 24 h.

Patients were asked to fill out a questionnaire before surgery and daily after surgery, until postoperative day (POD) 6. This patient diary contained questions regarding chewing gum use, oral intake, bowel movements, defaecation, gastrointestinal symptoms and visual analogue scale (VAS) pain score.

Surgeons or surgical residents were asked to fill out case record forms (CRF) with information regarding both patient and surgical characteristics, such as age, gender, body mass index (BMI), American Society of Anesthesiologists (ASA) score, medication use, smoking, operative procedure, postoperative course and postoperative complications (e.g. anastomotic leakage (AL), surgical site infection (SSI), fascial dehiscence, urinary tract infection (UTI) and pneumonia).

### Blood sample analysis

Peripheral blood samples was drawn from patients prior to the surgical procedure, and in the morning on POD1 and POD3. Measurements of plasma white blood cell count (WBC) and C-reactive protein (CRP) were performed by the hospital’s laboratory at these same time points. Blood samples were centrifuged and plasma was stored at −80 °C. Enzyme-linked immunosorbent assays (ELISAs) were performed according to instructions of the manufacturer (PeproTech Inc., Rocky Hill, USA) to quantify the concentration of the systemic inflammatory marker interleukin-6 (IL-6). A ratio of samples was calculated, through dividing the values of POD3 samples by those of the preoperative samples ($\mathrm{ratio}=\frac{\mathrm{cytokine}\ \mathrm{level}\ \mathrm{on}\ \mathrm{POD}3}{\mathrm{cytokine}\ \mathrm{level}\ \mathrm{before}\ \mathrm{surgery}}$).

### Outcome parameters

The primary study parameter was the time from surgery until the resolution of POI, defined as passage of faeces and toleration of solid food for at least 24 h [26]. Secondary endpoints included time to first flatus, hospitalisation length, postoperative (infectious) complications, postoperative mortality, postoperative opioid use, patient reported outcomes (e.g. pain score, nausea, regurgitations, vomiting, chewing gum use), inflammatory parameters (e.g. CRP, WBC and IL-6), blood pressure, body temperature and heart rate.

PPOI was defined as POI that was not resolved after POD5 or recurrent POI after an apparent resolution of POI. Diagnosis of PPOI was not made directly by the participating surgeons, but via retrospective review of the patient diary and medical record, to ensure objectiveness of the primary endpoint.

### Sample size calculation

According to Asao’s gum chewing experiment [24] and Flood’s nicotine trial [16], a sample size calculation was made, based on a mean POI time of 4.0 days in the chewing gum groups and an assumption of 2.6 days in the nicotine chewing gum group with a standard deviation of 1.5 days in both groups. In order to obtain a power of 80%, with an *α* level of 0.05, a number of 16 patients were needed in each group. As a dropout rate of 20% was expected, a total number of 40 patients (20 patients per group) were needed.

### Patient allocation

Randomisation was done with Microsoft Excel 2010 (Microsoft Corporation, Redmond, WA, USA) and results were placed and concealed in sequentially numbered, sealed, opaque envelopes by a person who was not connected to the trial. Patients were asked to participate by surgeons or specialised nurses who were involved in the trial. Patients were preoperatively randomised in a 1:1 design to either treatment with normal chewing gum or nicotine chewing gum. The allocated treatment was given to the patients by the nursing staff. Both patients and investigators were blinded for treatment allocation.

### Statistical analysis

Only patients who completed the full study period were analysed. Data analysis was carried out using the Statistical Package for the Social Sciences (SPSS Inc., Chicago, USA, version 21.0 for Windows). Demographic data were presented in *n* (%) and median (interquartile range [IQR]). Categorical variables were compared using the Fisher’s exact test. Continuous variables were compared using the Mann-Whitney *U* test.

## Results

**Fig. 2:**
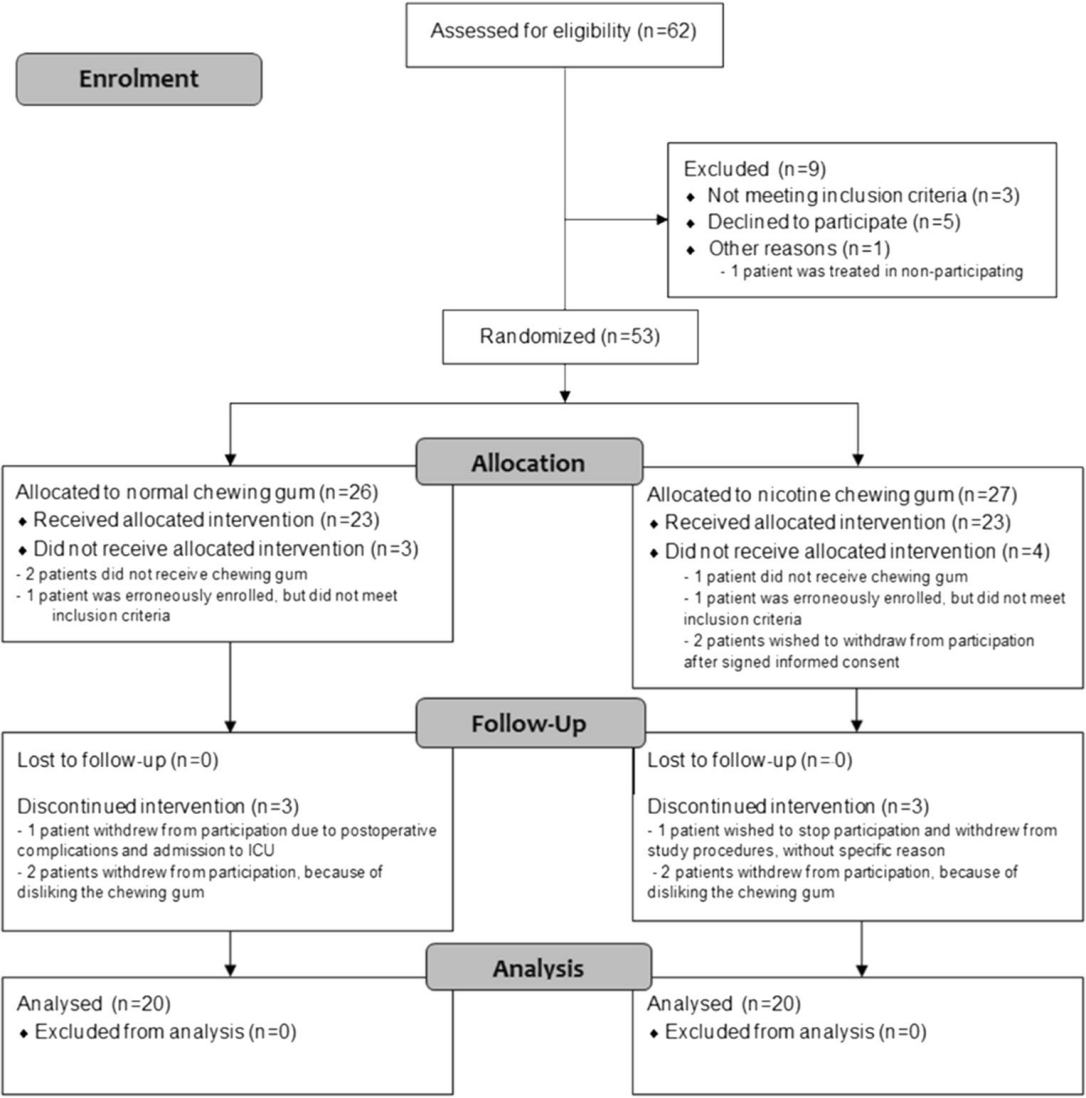
CONSORT flow diagram

In Fig. 2, the CONSORT flow diagram of the study is shown. Between January 29, 2015 and July 14, 2016, 62 patients were assessed for eligibility. Of these patients, 53 were randomly assigned to the normal chewing gum group or the nicotine chewing gum group. Two patients in each group withdrew from participation, because of disliking the chewing gum. One patient in the normal gum group was unable to continue treatment protocol, because of postoperative complications and ICU admission. The other patients withdrew for other reasons than disliking or being unable to chew the allocated gum. In total, 40 patients were included for data analysis.

Baseline patient and surgical characteristics were distributed evenly between both groups, without significant differences (Table 1). Urinary catheterisation failed in one patient in the normal gum group and one patient in the nicotine gum group required vasopressors for hemodynamic support during surgery. All patients who were admitted to the intensive care unit (ICU) directly after surgery were transferred to the surgical ward after 1 day.

**Table 1:** Baseline patient and surgical characteristics in treatment groups

	Normal gum (*n* = 20)	Nicotine gum (*n* = 20)
Patient characteristics		
Sex		
Male	13 (65)	14 (70)
Female	7 (35)	6 (30)
Age (years)	67.50 [60.75–74.75]	69.00 [62.50–70.00]
BMI (kg/m^2^)	26.91 [23.77–31.61]	25.02 [23.15–27.67]
Smoking	2 (10)	4 (20)
Diabetes mellitus	1 (5)	4 (20)
COPD	2 (10)	3 (15)
Cardiovascular disease	9 (45)	5 (25)
Corticosteroid use	3 (15)	1 (5)
Statin use	4 (20)	5 (25)
Neoadjuvant radiotherapy	1 (5)	0
Neoadjuvant chemoradiotherapy	1 (5)	0
Previous abdominal surgery	3 (15)	3 (15)
ASA classification		
ASA I	4 (20)	3 (15)
ASA II	13 (65)	14 (70)
ASA III	3 (15)	2 (10)
ASA IV	0	0
Surgical characteristics		
Type of procedure		
Low anterior resection	3 (15)	3 (15)
Left hemicolectomy	3 (15)	2 (10)
Right hemicolectomy	8 (40)	6 (30)
Sigmoidectomy	5 (25)	6 (30)
Subtotal colectomy	1 (5)	0
Transverse colon resection	0	3 (15)
Laparoscopic approach	20 (100)	16 (80)
Anastomotic technique		
End-to-end	4 (20)	2 (10)
End-to-side	1 (5)	1 (5)
Side-to-end	5 (25)	5 (25)
Side-to-side	10 (50)	11 (55)
Anastomotic configuration		
Stapled	12 (60)	13 (65)
Sutured	8 (40)	8 (40)
Protective ileostomy	2 (10)	2 (10)
Nasogastric tube	12 (60)	13 (65)
Intraoperative complications	1 (5)	1 (5)
>50-mL blood loss	5 (25)	9 (45)
Duration of surgery (min)	133 [101–176]	117 [109–150]
Postoperative ICU admission	1 (5)	2 (10)

Data are median [IQR] or *n* (%)

*BMI* body mass index, *ASA* American Society of Anesthesiologists classification, *ICU* intensive care unit

The time to primary endpoint (as defined earlier) as well as the time to first passage of faeces and flatus and length of stay (LOS) are given in Table 2. No statistically significant differences were found between groups. In both groups, six patients (30%) suffered from PPOI on or after POD6. Furthermore, there was no significant difference in the percentage of resolution of POI on POD1 to 5 (Table 3 and Online Resource Fig. S1). In a subgroup analysis in which all four open procedures were excluded, the time to primary endpoint in the nicotine gum group was shorter, but also not significantly different from the normal gum group (3.00 days [3.00–4.50] vs. 4.50 [3.00–7.25], *p* = 0.249).

**Table 2:** Time to primary endpoint, time to first passage of faeces and flatus, length of stay in days

	Normal gum (*n* = 20)	Nicotine gum (*n* = 20)	*p* value
Time to primary endpoint (days)	4.50 [3.00–7.25]	3.50 [3.00–4.25]	0.398
Time to first passage of faeces (days)	3.00 [1.75–5.00]	3.00 [1.75–4.00]	0.414
Time to first passage of flatus (days)	1.00 [1.00–2.25]	1.00 [1.00–1.00]	0.454
Length of stay (days)	5.50 [4.00–8.50]	4.50 [4.00–6.00]	0.738

Data are median [IQR]

**Table 3:** Resolution of POI

	Normal gum (*n* = 20)	Nicotine gum (*n* = 20)	*p* value
Resolution of POI			
POD1	0	0	–
POD2	0	2 (10)	0.487
POD3	6 (30)	9 (45)	0.515
POD4	11 (55)	13 (65)	0.748
POD5	14 (70)	14 (70)	1.000
POD6 or later	20 (100)	20 (100)	1.000

Data are *n* (%)

Six patients in the normal gum group and six in the nicotine group required a nasogastric tube during their postoperative stay. Three patients in the normal gum group and four in the nicotine gum group required total parental nutrition (TPN). Postoperative complications, reinterventions, readmissions and mortality during the first 30 days after surgery are given in Table 4. No differences were found between both treatment groups. Only one patient in the nicotine gum group had a short period of atrial fibrillation and overall, no myocardial infarction was seen. One patient in each treatment group required blood transfusion. One patient was readmitted because of anastomotic leakage and drainage of an intra-abdominal abscess, one patient was readmitted for adhesion ileus and one for observation of fever of unknown origin. One patient died during primary hospital stay, due to severe small bowel ischemia, caused by venous mesenteric thrombosis. Subgroup analysis for cases without intra-abdominal infectious complications during primary stay showed a time to primary endpoint of 4.00 days (3.00–5.50) vs. 4.50 (4.00–6.00) (*p* = 0.339) and LOS of 5.00 days (4.00–8.00) vs. 4.50 (4.00–6.00) (*p* = 0.673), for the normal gum and nicotine gum groups, respectively.

**Table 4:** Postoperative complications, reinterventions (surgical and/or radiological), readmissions and mortality (≤30 days)

	Normal gum (*n* = 20)	Nicotine gum (*n* = 20)	*p* value
Atrial fibrillation	0	1 (5)	1.000
Fascial dehiscence	0	0	–
Colorectal anastomotic leakage	2 (10)	0	0.487
Intra-abdominal abscess	1 (5)	0	1.000
Myocardial infarction	0	0	–
Pneumonia	1 (5)	0	1.000
Surgical site infection	4 (20)	2 (10)	0.661
Urinary retention	0	1 (5)	1.000
Urinary tract infection	2 (10)	1 (5)	1.000
Reinterventions (<30 days)	4 (20)	2 (10)	0.661
Readmissions (<30 days)	3 (15)	0	0.231
Mortality (<30 days)	0	1 (5)	1.000

Data are *n* (%)

### Postoperative opioid use

Fourteen patients in the normal gum group used oral opioids postoperatively, compared to 11 in the nicotine gum group (*p* = 0.514). Respectively, epidural opioids were used in 16 and 14 patients and a combination of oral and epidural opioids was used in 12 and 9 patients (*p* = 0.527). One patient in the normal group used a PCA pump and five patients in the nicotine gum group (*p* = 0.091). Patients in the nicotine gum group used epidural opioids for a significantly longer time (3.00 days [2.00–4.25] vs. 2.00 [1.00–.00], *p* = 0.006), but duration of oral opioid use did not differ between groups (1.00 day [0–3.50] vs. 1.00 [0–3.00], *p* = 0.740).

### Patient reported outcomes

Fifteen patients who received normal gum filled out their diary, as compared to 16 patients who received nicotine chewing gum (*p* = 1.000). Pain scores (VAS) were significantly lower in the nicotine gum group on POD3 (1.40 [0.50-] vs. 2.70 [1.50-], *p* = 0.007), but did not differ on the other postoperative days (Online Resource Fig. S2). No differences were found in patient reported nausea, vomiting, regurgitations, abdominal distension and appetite (Online Resource Fig. S3). Treatment compliance, as based on patient reported chewing gum use, is given in Online Resource Table S1.

### Inflammatory parameters

No significant differences were observed in IL-6 levels and white blood cell counts in preoperative samples and POD1 and three samples (Online Resource Fig. S4). CRP levels differed on POD1 in the normal and nicotine gum groups, respectively (71.50 mg/L [35.00–92.75] vs. 94.50 mg/L [58.50–128.25], *p* = 0.017), but no differences were found in preoperative and POD3 samples (Online resource Fig. S4b). None of the calculated ratios showed differences between both groups (Table 5). On none of the postoperative days, a statistically significant difference was found in systolic and diastolic blood pressure, and heart rate (Online Resource Fig. S5).

**Table 5:** Inflammatory parameters (Interleukin-6 (IL-6), C-reactive protein (CRP) and white blood cell (WBC) count

	Normal gum (*n* = 20)	Nicotine gum (*n* = 20)	*p* value
IL-6 (pg/mL)			
Preoperative	1088.95 [529.65–1680.70]	1108.40 [547.18–1732.38]	0.663
POD1	881.80 [516.90–2138.70]	1047.65 [752.53–1930.10]	0.883
POD3	959.00 [648.90–2043.60]	987.40 [518.38–2139.75]	0.940
Ratio	1.13 [0.99–1.54]	1.12 [0.89–1.24]	0.517
CRP (mg/L)			
Preoperative	2.60 [1.00–4.75]	3.70 [2.25–23.38]	0.089
POD1	71.50 [35.00–92.75]	94.50 [58.50–128.25]	0.017
POD3	99.50 [76.25–179.50]	151.00 [101.75–188.50]	0.180
Ratio	45.83 [19.70–83.68]	33.92 [6.59–79.10]	0.180
WBC count (×10^9^/L)			
Preoperative	7.10 [3.90–9.60]	6.60 [6.25–9.10]	0.477
POD1	12.30 [7.65–15.45]	12.30 [10.85–13.85]	0.865
POD3	8.50 [4.95–10.60]	9.00 [7.15–11.85]	0.583
Ratio	1.08 [0.95–1.59]	1.15 [0.99–1.77]	0.734

Data are median [IQR]

## Discussion

This study was the first to investigate the role of nicotine chewing gum for the prevention of postoperative ileus by assessing its clinical efficacy and safety in patients undergoing elective colorectal surgery. By performing this parallel-group, double-blind, randomised, controlled pilot study, it was not possible to prove the beneficial effect of nicotine chewing gum, as compared to normal chewing gum. We hypothesised that the combination of perioperative gum chewing, with the potential beneficial effects of nicotine, could improve the resolution of POI, but although the median time to primary endpoint seemed shorter in the nicotine gum group (3.50 days vs. 4.50), the difference was not statistically significant. Moreover, an equal number of patients (*n* = 6) in each group suffered from PPOI and LOS did not differ significantly between both groups. Open procedures are known to worsen POI outcomes in colorectal surgery as compared to the laparoscopic approach [27]. Since all four open procedures in this study were in the nicotine gum group, a subgroup analysis was performed in which these four procedures were excluded. This showed an improvement in median time to primary endpoint (3.50 vs. 3.00 days) and length of stay (4.50 vs. 4.00 days) in the nicotine gum group, but although these open procedures influenced the outcomes, they did not provide a complete explanation for the lack of efficacy, since a significant difference between both groups was still not found.

A limitation of this study might be the relatively small sample size of 40 patients in total. However, our sample size calculation was based on the results of Asao et al. [24] who showed significant effects of chewing gum in a total of 19 patients. With a larger sample size than we initially calculated, we might have had more power to make a better distinguishment between the effects of sham feeding with normal chewing gum and the hypothesised additional effects of sham feeding with nicotine chewing gum.

Experimental studies have shown that a specific α7-nAChR agonist (AR-R17779) ameliorates POI in rats and that stimulation of the α7-nAChR improves survival of sepsis in rats [11, 28]. Nevertheless, as clinical results of nicotine use for POI after colorectal surgery were still lacking, the second aim of this pilot study was to evaluate the safety of nicotine chewing gum for the purpose of preventing POI. Because of concerns of systemic effects induced by nicotine administration, particularly cardiovascular complications [29], we decided to use Nicorette® 2 mg. This relatively low dose might have potentially been another reason for the lack of efficacy of the nicotine chewing gum in this study. However, the use of Nicorette® 2 mg in a perioperative setting of elective colorectal surgery, which has not been described in the literature before, seems to be safe.

No myocardial infarctions were registered in this study and only one patient had one short period of atrial fibrillation. This patient did receive nicotine chewing gum, but was known to have had previous episodes of paroxysmal atrial fibrillation. These findings are consistent with a previously published Cochrane Review, concluding that there is no evidence that nicotine replacement therapy increases the risk of heart attacks [30]. Moreover, apart from cardiovascular complications, no differences were found in major and minor postoperative complications, reinterventions, readmissions and mortality between the normal and nicotine chewing gum groups. Overall, only one patient—enrolled in the nicotine chewing gum group—died during primary hospital stay on the 15th postoperative day. We did not consider usage of the nicotine chewing gum related to the cause of death, which was a result of intestinal ischemia caused by venous mesenteric thrombosis.

To combine the potential benefits of sham feeding with chewing gum and nicotine, we chose to use nicotine chewing gum in this pilot study. The reason being that this facilitated the possibility for a simple blinded comparison with normal chewing gum. However, a limitation of nicotine administration through chewing gum is that a sufficient release of nicotine is dependent on treatment compliance of the patient. All patients in this study were asked to report their use of chewing gum in the patient diary and it can be concluded that compliance to chewing the allocated gum is decreasing in the first three postoperative days. Conceivably, a more constant way of nicotine administration, such as the nicotine patches which Habib et al. [15] used in their study, might have given a more continuous release of nicotine.

If indeed the effective dose of nicotine would be too low in some patients, either due to low administered dose, insufficient exposure to the nicotine chewing gum or both, this might account for the absence of significant differences between the measured clinical and inflammatory parameters in both groups.

Il-6 and CRP levels, as well as WBC, were analysed in venous blood samples, as markers of the immune response, because it was hypothesised that this response would be less pronounced in patients in the nicotine chewing gum group. Overall lower levels of any of the three inflammatory parameters (IL-6, CRP and WBC) were not seen in this group and no differences were found, when compared to the normal chewing gum group, except for a significant difference of CRP levels on POD1. Sparreboom et al. [31] have concluded in their meta-analysis that levels of pro-inflammatory cytokines, such as IL-6, were higher in peritoneal samples as compared to serum samples after colorectal surgery, which might also explain why significant changes and differences were not detected in our serum samples. Moreover, postoperative infectious complications, such as surgical site infections and pneumonia, could have affected the levels of these inflammatory parameters in both groups.

No differences in patient reported outcomes, such as nausea, vomiting, regurgitations, abdominal distension and appetite, were found. However, patient reported pain scores were significantly lower in the nicotine gum group as compared to the normal gum group on POD3 (1.40 [0.50-] vs. 2.70 [1.50-], *p* = 0.007). Although promising, this difference could partially be explained by the fact that patients in this group received epidural opioids for a significantly longer period of time (3.00 days [2.00–4.25] vs. 2.00 [1.00–.00], *p* = 0.006). However, the exact reason for a longer use of epidural opioids remains uncertain, since the decision to stop epidural anaesthesia in this study was made by the anaesthesiologist, who was blinded for patient allocation, with the aim to stop as early as possible, and preferably on or before POD2–3. These decisions were not registered prospectively. Furthermore, no significant differences in patient outcomes were found between both groups which might explain the extended requirement for epidural opioids in the nicotine gum group.

In conclusion, this study is the first to evaluate the potential beneficial role of nicotine chewing gum for another purpose than NRT in a randomised and double-blind clinical setting. Although the hypothesised potential benefits of nicotine chewing gum, as a cheap and readily available treatment option, seemed promising, no evident beneficial effects were found. This might be attributed to the sample size, the dose of the nicotine chewing gum and insufficient patient compliance to the allocated chewing gum. People in the nicotine chewing gum group seemed to experience less pain in the first three postoperative days, but a difference could only be proven on POD3. Therefore, more data on the effects of nicotine gum on bowel recovery after surgery are awaited [32]. Furthermore, this study provides positive new insights on the safety of nicotine chewing in the setting of patients undergoing elective colorectal surgery. Future research should focus on other means of nicotine administration to patients undergoing colorectal surgery (e.g. patches), whether or not combined with normal chewing gum, and in higher doses, to further assess its effects on gastrointestinal recovery after colorectal surgery.

## Electronic supplementary material

Table S1 (DOCX 82 kb)

Supplementary Figure S1 Resolution of POI (**a**, **b**) (GIF 2 kb)

High Resolution Image (TIFF 2879 kb)

Supplementary Figure S2 Postoperative patient reported pain scores (Visual Analogue Scale) (GIF 1 kb)

High Resolution Image (TIFF 2164 kb)

Supplementary Figure S3 Postoperative patient reported outcomes: (**a**) Nausea, (**b**) Vomiting, (**c**) Regurgitations, (**d**) Abdominal distension, (**e**) Appetite (GIF 3 kb)

High Resolution Image (TIFF 4077 kb)

Supplementary Figure S4 Inflammatory parameters (**a**) Interleukin-6 (IL-6) levels, (**b**) C-reactive protein (CRP) levels, (**c**) white blood cell count (GIF 4 kb)

High Resolution Image (TIFF 3699 kb)

Supplementary Figure S5 Postoperative outcomes: (**a**) Systolic blood pressure, (**b**) Diastolic blood pressure, (**c**) Heart rate. Normal gum = ● (dots), nicotine gum = ■ (squares) (GIF 2 kb)

High Resolution Image (TIFF 2741 kb)
