# Supplementary figures and images for: Nicotine chewing gum for the prevention of postoperative ileus after colorectal surgery: a multicenter, double-blind, randomised, controlled pilot study
Source: Int J Colorectal Dis. 2017 Jun 28;32(9):1267–75. doi: 10.1007/s00384-017-2839-z (PMC5554272; doi:10.1007/s00384-017-2839-z)

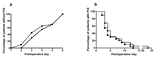

Supplement: Supplementary file 2 — Resolution of POI (a, b) (GIF 2 kb) [file 384_2017_2839_Fig3_ESM.gif]

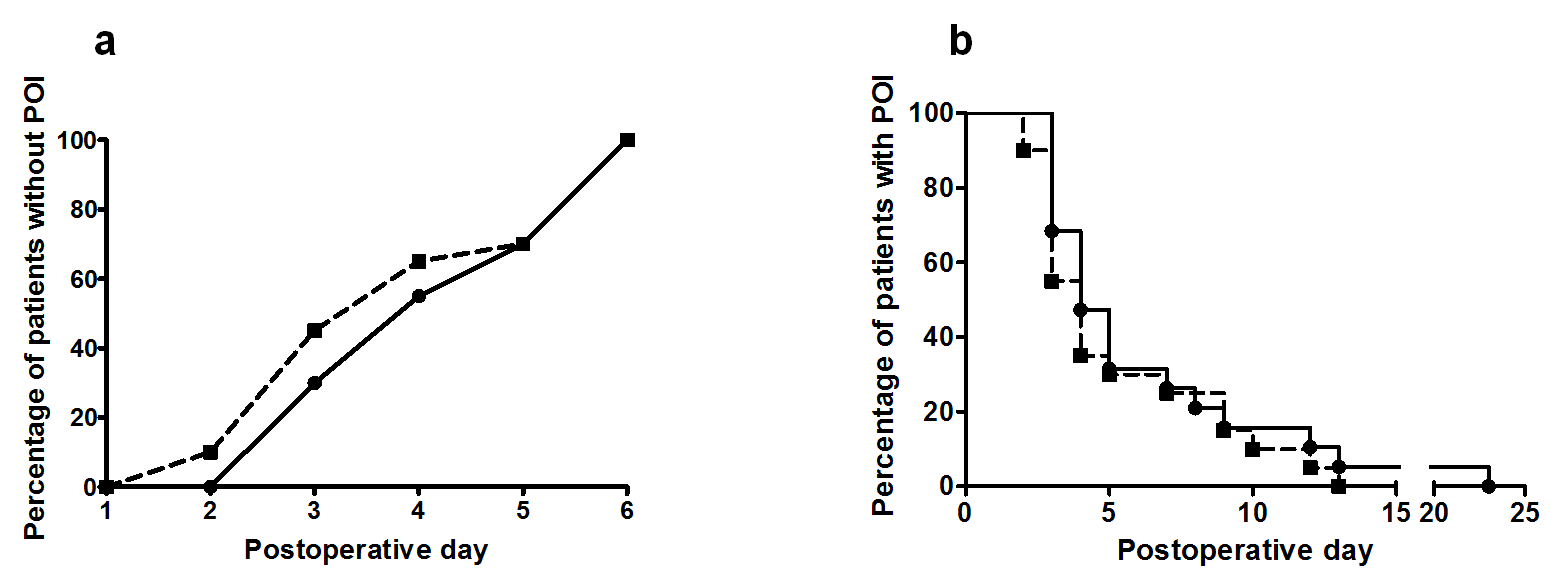

Supplement: Supplementary file 3 — High Resolution Image (TIFF 2879 kb) [file 384_2017_2839_MOESM2_ESM.tif]

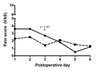

Supplement: Supplementary file 4 — Postoperative patient reported pain scores (Visual Analogue Scale) (GIF 1 kb) [file 384_2017_2839_Fig4_ESM.gif]

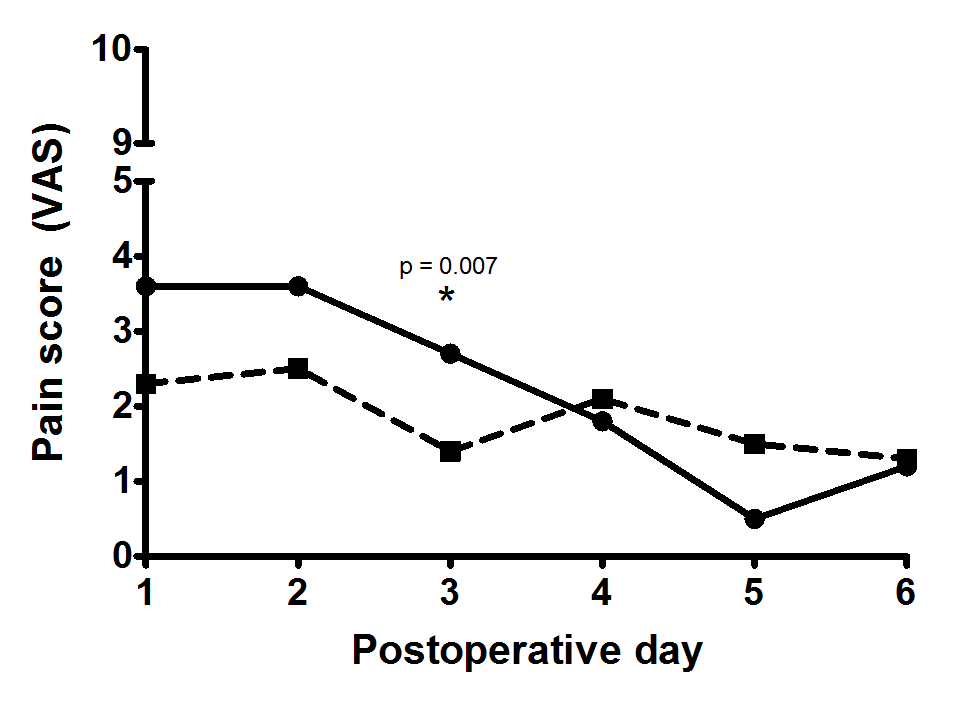

Supplement: Supplementary file 5 — High Resolution Image (TIFF 2164 kb) [file 384_2017_2839_MOESM3_ESM.tif]

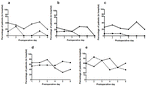

Supplement: Supplementary file 6 — Postoperative patient reported outcomes: (a) Nausea, (b) Vomiting, (c) Regurgitations, (d) Abdominal distension, (e) Appetite (GIF 3 kb) [file 384_2017_2839_Fig5_ESM.gif]

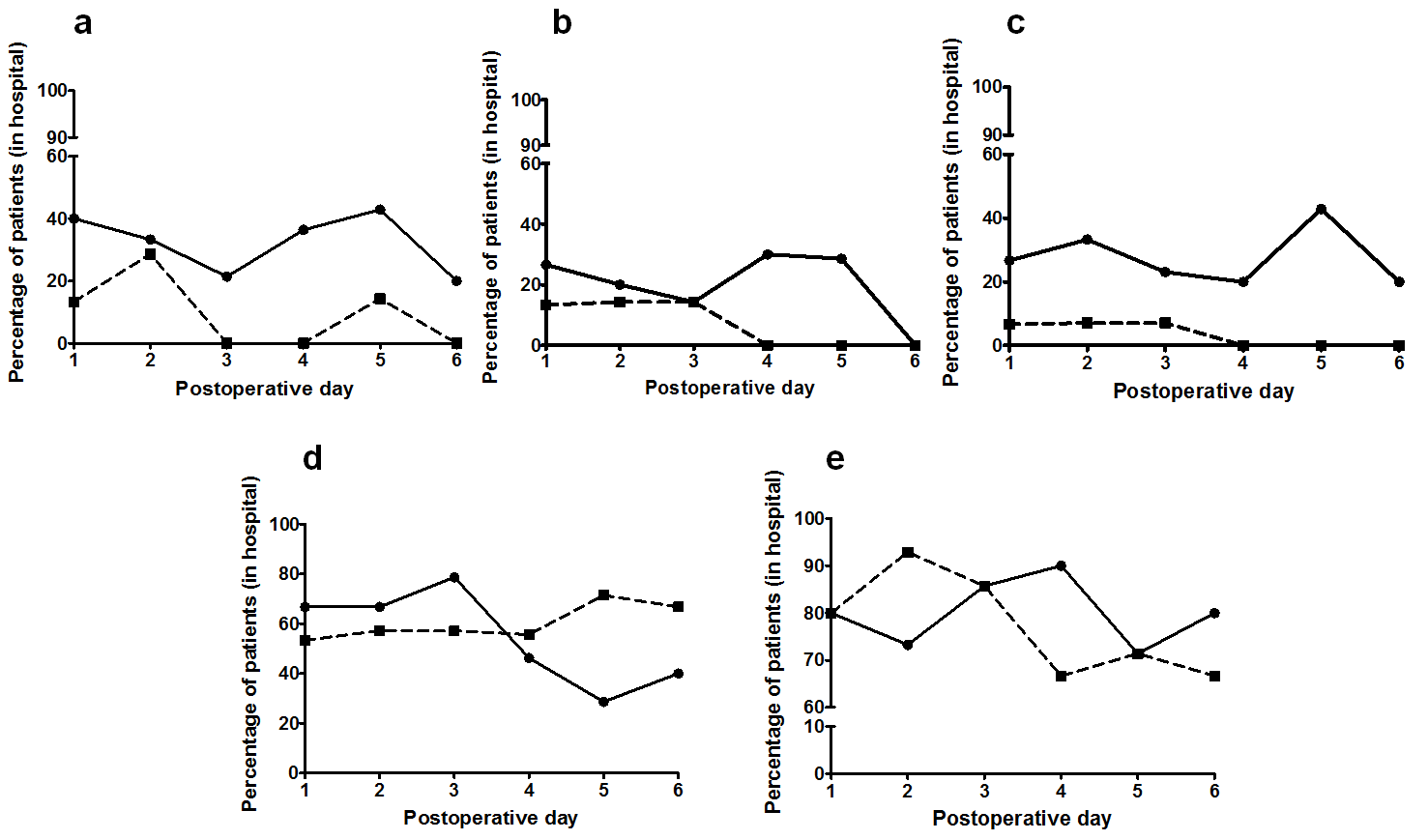

Supplement: Supplementary file 7 — High Resolution Image (TIFF 4077 kb) [file 384_2017_2839_MOESM4_ESM.tif]

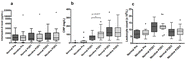

Supplement: Supplementary file 8 — Inflammatory parameters (a) Interleukin-6 (IL-6) levels, (b) C-reactive protein (CRP) levels, (c) white blood cell count (GIF 4 kb) [file 384_2017_2839_Fig6_ESM.gif]

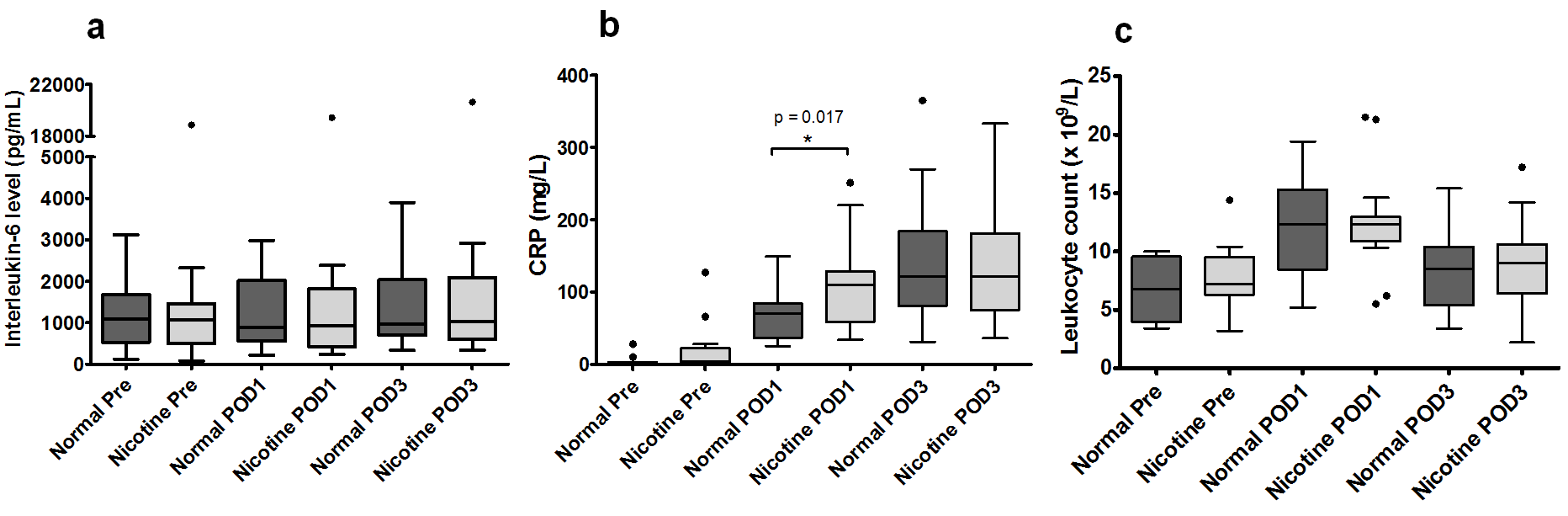

Supplement: Supplementary file 9 — High Resolution Image (TIFF 3699 kb) [file 384_2017_2839_MOESM5_ESM.tif]

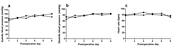

Supplement: Supplementary file 10 — Postoperative outcomes: (a) Systolic blood pressure, (b) Diastolic blood pressure, (c) Heart rate. Normal gum = ● (dots), nicotine gum = ■ (squares) (GIF 2 kb) [file 384_2017_2839_Fig7_ESM.gif]

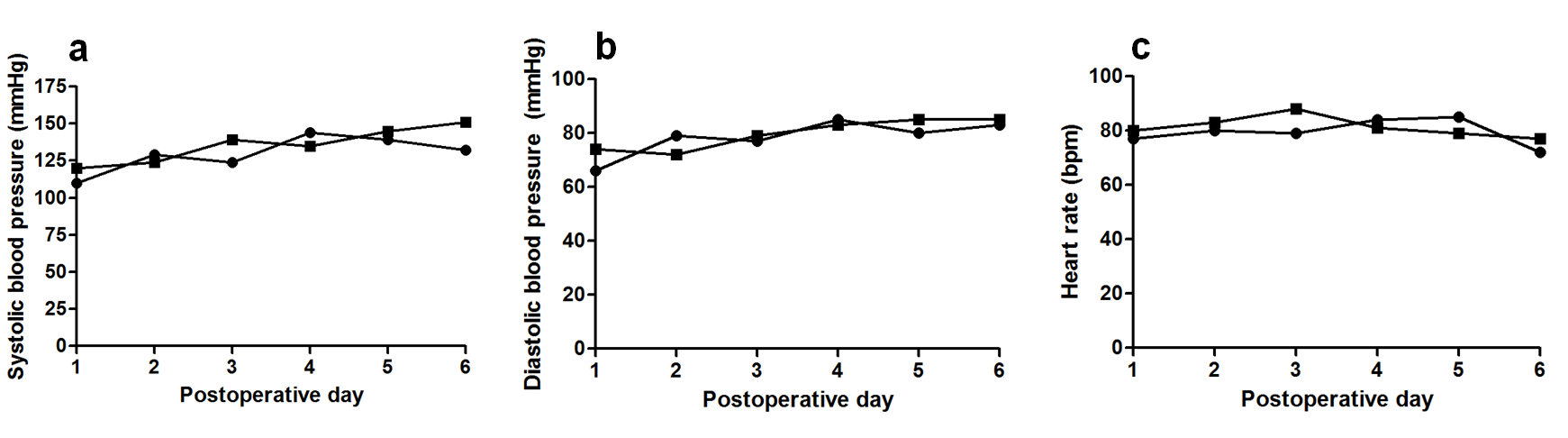

Supplement: Supplementary file 11 — High Resolution Image (TIFF 2741 kb) [file 384_2017_2839_MOESM6_ESM.tif]
