# Supplementary material for: Nicotine chewing gum for the prevention of postoperative ileus after colorectal surgery: a multicenter, double-blind, randomised, controlled pilot study
Source: Int J Colorectal Dis. 2017 Jun 28;32(9):1267–75. doi: 10.1007/s00384-017-2839-z (PMC5554272; doi:10.1007/s00384-017-2839-z)
Supplement: Supplementary file 2 [file 384_2017_2839_MOESM1_ESM.docx]

	Normal gum (n = 20)	Nicotine gum (n = 20)
POD1		
As in protocol	7 (35)	9 (45)
Less than protocol	5 (25)	4 (20)
More than protocol	2 (10)	2 (10)
Not reported	6 (30)	5 (25)
POD2		
As in protocol	7 (35)	7 (35)
Less than protocol	5 (25)	8 (40)
More than protocol	1 (5)	0
Not reported	7 (35)	5 (25)
POD3		
As in protocol	3 (15)	3 (15)
Less than protocol	11 (55)	13 (65)
More than protocol	0	0
Not reported	6 (30)	4 (20)

Data are n (%)

Online Resource Supplementary Table 1 Patient reported compliance to chewing gum
